# Theoretical study of diffusive model of HIV-1 infection and its analytical solution

**DOI:** 10.1371/journal.pone.0283659

**Published:** 2023-11-10

**Authors:** Noufe H. Aljahdaly, R. A. Alharbey, Ahlam S. Aljohani

**Affiliations:** 1 Mathematics Department, Faculty of Sciences and Arts, King Abdulaziz University, Rabigh, Saudi Arabia; 2 Department of Mathematics, Faculty of Science, Al-Sulymania Womenans Campus, King AbdulAziz University, Jeddah, Saudi Arabia; Shanxi University, CHINA

## Abstract

T his article studied a mathematical model for the diffusive human immunodeficiency virus-type 1 (HIV-1) infection combining with stem cell therapy. The HIV-1 infection is a chronic disease and the viral replication continues if the patient stopes use the antiretroviral therapy (cART). Therefore, it is important to seek the cure of HIV-1 infection and some medical trials showed the cure by stem cell therapy and there are others failure to achieve the cure of HIV-1 with same treatments. The novelty of this paper is constructing a mathematical model with adding diffusion terms to study the effect of spread of virus and other cells in the body. Theoretical analysis such as boundedness, positivity, stability (local/global) of the HIV-1 model is presented. The model is solved analytically by the tanh expansion method. The results show that the tanh expansion method is a very useful technique, that can give a good prediction of the effect of stem cell therapy on infected cells on the spread of the virus. The results further demonstrated that the best way to control the disease is by limiting the spread of the virus; more so than the spread of other components.

## Introduction

Epidemics are mostly caused by human activities that bring about environmental changes [1]. Microorganisms adapt and change, resulting in the emergence of devastating diseases, such as AIDS, Ebola, malaria, and COVID-19 which have resulted in very large numbers of deaths. The human immunodeficiency virus (HIV) remains a major public health concern since, 1981, when HIV/AIDS was diagnosed as a new illness [2–5]. According to the World Health Organization, more than 36 million people have died due to complications resulting from HIV infection.

HIV is linked to the weakening and ultimate destruction of immune system mostly due to the depletion of CD4^+^ *T* -cells [6, 7]. The HIV infection begins as soon as the virus enters the host. The CD4^+^ cells which are receptor-positive (the majority of which are lymphocytes), are afflicted. The HIV virus, on entering a cell can either stay quiescent or multiply in a controlled manner, but in many cases can start rapid reproduction causing the infected cell to die. The virus is latent in most lymphocytes, and infection greatly reduces the number of cells in both the tissues and the blood available to deal with, secondary infection bacterial. As a result, HIV-positive individuals have a high death rate [2].

Several treatments have been proposed to improve the quality of life of HIV patients, including antiretroviral medication and chemotherapy [8]. Among other advances in oncology, researchers have inspired new treatment options for HIV since the early 1980s, one of which is stem cell transplantation [9]. The stem cell (SC) is a type of cell that has the remarkable capacity of regenerating it selves [10]. Human pluripotent stem cells include human embryonic stem cells (hESC) and human-induced pluripotent stem cells (hiPSC). In 1998, a case study of two HIV-positive individuals who received allogeneic SC transplantation was published and showed that the therapy causes viral decay and the relief of symptoms [11]. A second case study was published in Nature in 2019, which showed that on HIV-1 patient, having under gone SC treatment, had no recurrence of the infection after 18 months [12].

CD4^+^ −*T* lymphocytes play a key role in modulating the immune response, by secreting specific cytokines after activation and differentiation into different effector subtypes. These CD4^+^ *T*-cells have a variety of roles, including the activation of innate immune system cells, B lymphocytes, cytotoxic *T*-cells, and non-immune cells, as well as the inhibition of the immune response [13].

Because of the difficulties of identifying healthy matched donors, and the high cost of the treatment, stem cell therapy as an option is fairly limited [14].

The stem cell exists in a specific niche which enables it to undergo self-renewing divisions. It also generates differentiated cells via a population of committed but still dividing transit-amplifying cells. Not all stem cell types generate multiple types of differentiated cells. The infection is the invasion and growth of germs in the body. The germs may be bacteria, viruses, yeast, fungi, or other microorganisms. Infections can begin anywhere in the body and may spread all through it. An infection can cause fever and other health problems, depending on where it occurs in the body, [15].

Our understanding of AIDS relies heavily on mathematical models of HIV-1 infection [16]. Many such models have been constructed and examined, in order to have a better understanding of HIV dynamics before and during therapy. For example, a single patient with on HIV-1 infection was first presented by Perelson et al. in 1996. The team, used a basic virology model to study the interaction between viruses and CD4^+^ T-cells. There are three variables in the model to describe virus infection which are uninfected CD4^+^ T-cells; productively-infected *T* cells; and free virus. Some more complicated models have considered disease progression, antiretroviral therapy, and vaccine development [17–19].

However, the first numerical study investigating HIV-1 infection and stem cells was iconducted by Alqudah and Aljahdaly [12]. The type of calculus used is the branch that generalizes the derivative of a function to an arbitrary order. Applications of calculus have proliferated in the past few decades [20]. This is because the resulting models, in contrast to integer-order models, provide a more realistic depiction of system memory and genetic features [21]. Aljahdaly and Alharbey initially have devised a mathematical model for HIV-1 infection with stem cell therapy [22] then modified the model to include the effect of immune system cell response [23].

In the above instances, the models are classified as a system of ordinary differential equations. The novelty of this paper is that the model incorporates the relationship between the diffusion of the SC, the virus, and the infected and uninfected cells. In this instance, the model becomes a system of partial differential equations. The tanh expansion approach, which has been introduced and tested in this study, is one of the most direct and effective algebraic methods for obtaining accurate solutions to nonlinear diffusion equations. The tanh expansion method is an analytical method which is able to construct the solution as a polynomial of tanh function, without the need of initial or boundary conditions. The Several researchers have used this strategy to find solutions to various PDEs up to the time of writing [24–26].

In this paper, we propose the mathematical model that describes the concentration of uninfected CD4^+^T -cells, infected CD4^+^T- cells, HIV-1 in the blood, and stem cells; whilst considering the diffusion effect for all components. The paper is organized as follows: In Section 2, presents the mathematical model; Section 3 presents a comprehensive theoretical study including existence, uniqueness, positivity, boundedness and stability; Section 4 shows the analytical solutions; and Section 5 presents the discussion of the solution dynamic and followed by conclusions in Section 6.

## The mathematical mode

**Fig 1:**
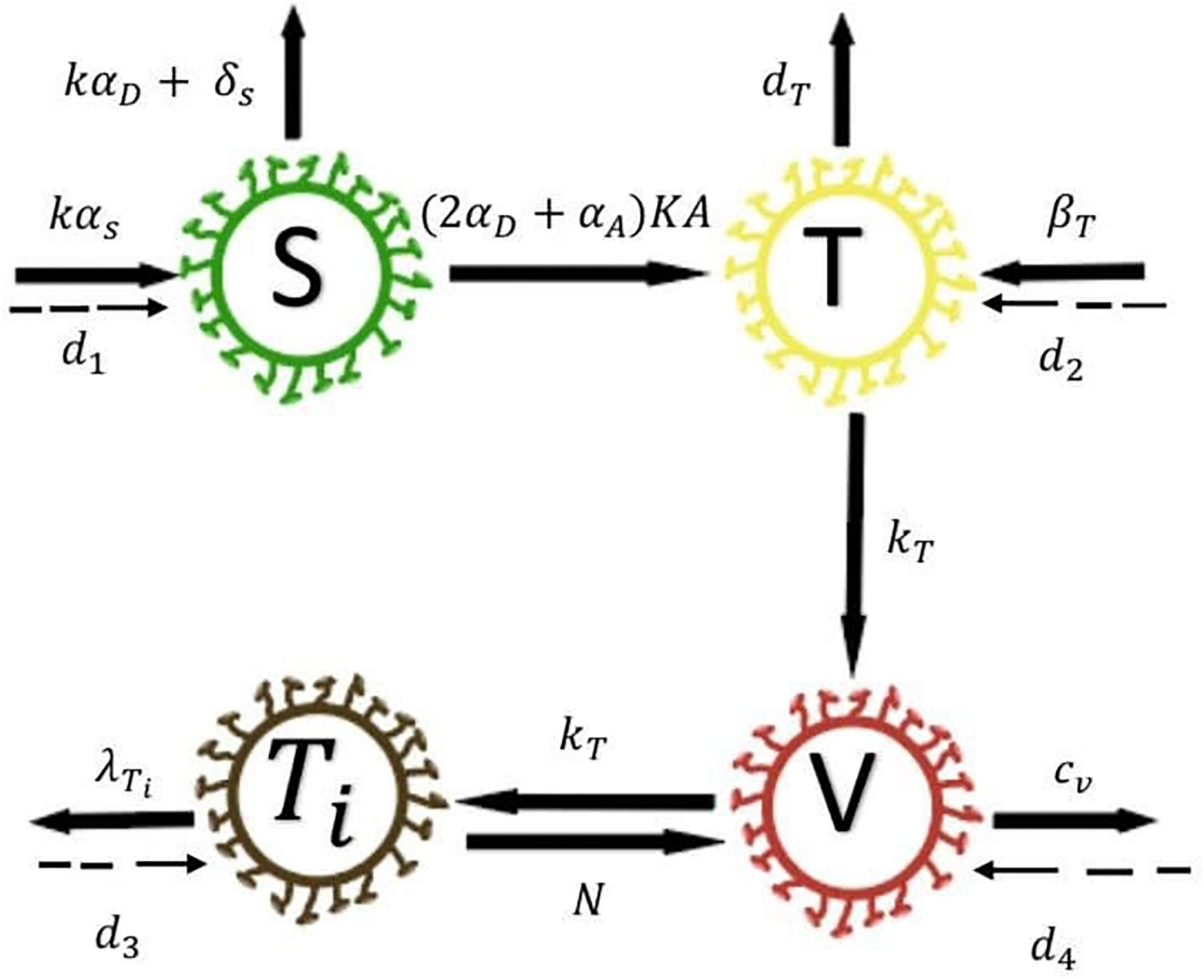
The diagram of the mathematical model (1).

The first mathematical model for predicting the relationship between HIV-1 infection and stem cell therapy, comprises the interaction of four components, unaffected CD4^+^T-cells (*T*), infected CD4^+^T-cells (*T*_*i*_), virus (*V*) and stem cells (*S*) (see Fig 1) [22]. The number of cells (or virus) in a microliter (cells/*μ*l) or (virus/*μ*l) is used to assess the concentration of cells and virus in blood fluid. In this paper, the model in ([22]) is modified by adding the diffusion terms for each component. Thus, we obtain the following system of time-partial differential equations:

$$\begin{array}{cc} \frac{\partial S(x,t)}{\partial t}= & \left(k\left(\alpha_{s}-\alpha_{D}\right)-\delta_{s}\right)S\left(x,t\right)+d_{1}\frac{\partial^{2}S\left(x,t\right)}{\partial x^{2}},\\ \frac{\partial T{\left(x,t\right)}_{}}{\partial t}= & \beta_{T}-d_{T}T\left(x,t\right)+\left(2\alpha_{D}+\alpha_{A}\right)kAS\left(t\right)+r_{T}T\left(x,t\right)(1-\frac{T(x,t)}{T_{max}})\\ & -k_{T}T\left(x,t\right)V\left(x,t\right)+d_{2}\frac{\partial^{2}T\left(x,t\right)}{\partial x^{2}},\\ \frac{\partial T_{i}\left(x,t\right)}{\partial t}= & k_{T}T\left(x,t\right)V\left(x,t\right)-\lambda_{T_{i}}T_{i}\left(x,t\right)+d_{3}\frac{\partial^{2}T_{i}\left(x,t\right)}{\partial x^{2}},\\ \frac{\partial V(x,t)}{\partial t}= & N\lambda_{T_{i}}T_{i}\left(x,t\right)-c_{v}V\left(x,t\right)+d_{4}\frac{\partial^{2}V\left(x,t\right)}{\partial x^{2}}, \end{array}$$ (1)

where *d*_1_, *d*_2_, *d*_3_, *d*_4_ are the diffusion term of *S*, *T*, *T*_*i*_, and *V*, respectively. The $\frac{\partial^{2}}{\partial x^{2}}$ is a diffusion operator and the parameters are described in Table 1.

**Table 1:** Descriptions and values of the parameters in the model (1) [12, 22].

Initial/Parameters	Description	Estimated Values
*S*_0_	Before differentiation and production of *T* cells the transplanting SCs were concentrated.	18 cells/*μ*l
*T*_0_	Uninfected CD4^+^T-cell concentration in the blood at the start.	900 cells/*μ*l
$T_{i_{0}}$	The initial concentration of infected CD4^+^T-cells was determined after the patient’s blood was screened for the first time.	100 cells/*μ*l
*V*_0_	When a virus’s concentration is high enough it can kill uninfected cells.	10^−6^ virus/*μ*l
*β*_*T*_	Uninfected CD4^+^T-cell production rate	0.17 cells/*μ*l.day
*d*_*T*_	Uninfected CD4^+^ T-cell death rate	0.01/day
*k*_*T*_	CD4^+^T-cell infection rate	6.5 × 10^−4^ Virus /*μ*l.day
$\lambda_{T_{i}}$	Infected CD4^+^T-cells’ mortality rate	0.39/day
*k*	Stem cell division rate	0.035/day
*δ*_*s*_	Stem cell death rate	0.03/day
*c*_*v*_	virus’s clearance rate	3 /day
*α*_*s*_	Asymmetric self-renewal probability	0.21/day
*α*_*D*_	At a rate of division into undifferentiated and differentiated cells	0.16 /day
*α*_*A*_	Symmetric self-renewal probability	0.6 /day
*A*	Factor of amplification	0.5
*N*	The number of virus particle produced by each *T*_*i*_ cell	10
*r*_*T*_	mitotic rate of T	3
*T*_*max*_	maximum population level of T.	1500

## Theoretical analysis

This study required a comprehensive investigation of our mathematical HIV-1 model, by examining its positivity, boundedness and stability (local and global).

1. Positivity solution and boundedness solution
  **Theorem 1** *If* $\left(S\left(x,t\right),T\left(x,t\right),T_{i}\left(x,t\right),V\left(x,t\right)\right)\in R_{4}^{+}$ , *then the following set is bounded and positively invariant*,
  $$\begin{array}{cc} \Omega = & \{\left(\bar{S}\left(\xi \right),\bar{T}\left(\xi \right),{\bar{T}}_{i}\left(\xi \right),\bar{V}\left(\xi \right)\right):0\leq \bar{S}\leq S_{o},0\leq \bar{T}+{\bar{T}}_{i}\leq \frac{\left(\beta_{T}+\left(2\alpha_{D}+\alpha_{A}\right)kA{\bar{S}}_{o}\right)}{\gamma },\\ & \bar{V}\leq \frac{N\lambda_{T_{i}}}{c_{v}}\frac{\left(\beta_{T}+\left(2\alpha_{D}+\alpha_{A}\right)kA{\bar{S}}_{o}\right)}{\gamma }\}. \end{array}$$
  *where* $\left(\bar{S}\left(\xi \right),\bar{T}\left(\xi \right),{\bar{T}}_{i}\left(\xi \right),\bar{V}\left(\xi \right)\right)=\left(S\left(x,t\right),T\left(x,t\right),T_{i}\left(x,t\right),V\left(x,t\right)\right),\xi =mx+ct$, $\gamma =min\{\frac{d_{T}}{c},\frac{\left(1-N\right)\lambda_{T_{i}}}{c},\frac{c_{v}}{c}\}$, *where c is the wave speed and m is the frequency number*.
**Proof**
  Assume that (*S*, *T*, *T*_*i*_, *V*) ∈ *C*[*a*, *b*] and *D*_*t*_(*S*, *T*, *T*_*i*_, *V*) ∈ *C*(*a*, *b*]. We note from Eq 1 the following:
  $$\begin{array}{cc} D_{t}{S|}_{S=0}= & 0,\geq 0,\\ D_{t}{T|}_{T=0}= & \beta_{T}+\left(2\alpha_{D}+\alpha_{A}\right)kAS\left(x,t\right)\geq 0,\\ D_{t}T_{i}{|}_{T_{i}=0}= & k_{T}T\left(x,t\right)V\left(x,t\right)\geq 0,\\ D_{t}{V|}_{V=0}= & N\lambda_{T_{i}}T_{i}\left(x,t\right)\geq 0. \end{array}$$
  Since *D*_*t*_(*S*(*x*, *t*), *T*(*x*, *t*), *T*_*i*_(*x*, *t*), *V*(*x*, *t*)) ≥ 0, ∀(*t*) ∈ (*a*, *b*), then *S*, *T*, *T*_*i*_, *V* are nondecreasing functions at each *t* ∈ [*a*, *b*]. Therefore, the solutions for *S*, *T*, *T*_*i*_, *V* are non-negative solutions. In order to study the boundedness, we convert the system to ODE using traveling wave transformation. Assume $\xi =mx+ct,\bar{S}(\xi )=S(x,t),\bar{T}(\xi )=T(x,t),{\bar{T}}_{i}(\xi )=T_{i}(x,t),\bar{V}(\xi )=V(x,t)$, thus the system of Eq (1) converts to the following
  $$\begin{array}{cc} \frac{d\bar{S}\left(\xi \right)}{d\xi }= & \frac{1}{c}(\left(k\left(\alpha_{s}-\alpha_{D}\right)-\delta_{s}\right)\bar{S}\left(\xi \right)+d_{1}m^{2}\frac{d^{2}\bar{S}\left(\xi \right)}{d\xi^{2}}),\\ \frac{d\bar{T}\left(\xi \right)}{d\xi }= & \frac{1}{c}(\beta_{T}-d_{T}\bar{T}\left(\xi \right)+\left(2\alpha_{D}+\alpha_{A}\right)kA\bar{S}\left(\xi \right)+\\ & r_{T}\bar{T}\left(\xi \right)(1-\frac{\bar{T}\left(\xi \right)}{T_{max}})-k_{T}\bar{T}\left(\xi \right)\bar{V}\left(\xi \right)+m^{2}d_{2}\frac{d^{2}\bar{T}\left(\xi \right)}{d\xi^{2}}),\\ \frac{d{\bar{T}}_{i}\left(\xi \right)}{d\xi }= & \frac{1}{c}(k_{T}\bar{T}\left(\xi \right)\bar{V}\left(\xi \right)-\lambda_{T_{i}}{\bar{T}}_{i}\left(\xi \right)+m^{2}d_{3}\frac{d^{2}{\bar{T}}_{i}\left(\xi \right)}{d\xi^{2}}),\\ \frac{d\bar{V}\left(\xi \right)}{d\xi }= & \frac{1}{c}(N\lambda_{{\bar{T}}_{i}}{\bar{T}}_{i}\left(\xi \right)-c_{v}\bar{V}\left(\xi \right)+m^{2}d_{4}\frac{d^{2}\bar{V}\left(\xi \right)}{d\xi^{2}}). \end{array}$$ (2)
  The solution of $\bar{S}$ under the boundary conditions of boundedness is
  $$\bar{S}\left(\xi \right)=C_{1}e^{\frac{1}{2}(\frac{c}{d_{1}m^{2}}-\sqrt{\frac{c^{2}}{d_{1}^{2}m^{4}}-\frac{4(k\left(\alpha_{s}-\alpha_{D}\right)-\delta_{s})}{d_{1}m^{2}}})\xi },$$
  where, *C*_1_ is the constant obtained by boundary conditions. Thus, $\bar{S}$ is bounded for all *ξ*, if (*k*(*α*_*s*_ − *α*_*D*_) − *δ*_*s*_ ≤ 0. Therefore, $\bar{S}\left(\xi \right)\leq {\bar{S}}_{o}$ for all *ξ*. From the system 2, we have
  $$\begin{array}{cc} \frac{d(\bar{T}\left(\xi \right)+{\bar{T}}_{i}\left(\xi \right))}{d\xi }= & \frac{\beta_{T}}{c}-\frac{d_{T}}{c}\bar{T}\left(\xi \right)+\frac{(2\alpha_{D}+\alpha_{A})kA}{c}\bar{S}\left(\xi \right)-\frac{\lambda_{T_{i}}}{c}{\bar{T}}_{i}\left(\xi \right)\\ & +\frac{r_{T}}{c}\bar{T}\left(\xi \right)(1-\frac{T(\xi )}{T_{max}})+\frac{m^{2}}{c}\frac{d^{2}\left(d_{2}\bar{T}\left(\xi \right)+d_{3}\bar{T_{i}}\left(\xi \right)\right)}{d\xi^{2}} \end{array}$$
  using $\bar{S}\left(\xi \right)\leq S_{o}$, $\bar{T}\left(\xi \right)\leq T_{max}$ for all *ξ* and using the theory of second derivative test that if $\bar{T}\left(\xi \right)$ and ${\bar{T}}_{i}\left(\xi \right)$ have local maximum at *c* if $\bar{T}\left(c\right)=0$ and ${\bar{T}}_{i}\left(c\right)=0$ and
  $$\frac{d^{2}\left(d_{2}\bar{T}\left(\xi \right)+d_{3}\bar{T_{i}}\left(\xi \right)\right)}{d\xi^{2}}\leq \frac{d^{2}\left(d_{2}\bar{T}\left(c\right)+d_{3}\bar{T_{i}}\left(c\right)\right)}{d\xi^{2}}<0.$$
  Thus,
  $$\frac{d(\bar{T}\left(\xi \right)+{\bar{T}}_{i}\left(\xi \right))}{d\xi }\leq \frac{\beta_{T}}{c}-\frac{d_{T}}{c}\bar{T}\left(\xi \right)+\frac{(2\alpha_{D}+\alpha_{A})kA}{c}{\bar{S}}_{o}-\frac{\lambda_{T_{i}}}{c}{\bar{T}}_{i}\left(\xi \right).$$
  Take $\gamma =min(d_{T},\lambda_{T_{i}}),$ then
  $$\frac{d(\bar{T}\left(\xi \right)+{\bar{T}}_{i}\left(\xi \right))}{d\xi }\leq (\frac{\beta_{T}}{c}+\frac{(2\alpha_{D}+\alpha_{A})kA}{c}{\bar{S}}_{o})-\frac{\gamma }{c}(\bar{T}\left(\xi \right)+{\bar{T}}_{i}\left(\xi \right)),$$
  since
  $$\underset{\xi \to \infty }{\text{lim}}\;\text{sup}\frac{d(\bar{T}\left(\xi \right)+{\bar{T}}_{i}\left(\xi \right))}{d\xi }\leq \frac{\left(\beta_{T}+\left(2\alpha_{D}+\alpha_{A}\right)kA{\bar{S}}_{o}\right)}{\gamma }.$$
  Therefore,
  $$\bar{T}\left(\xi \right)+{\bar{T}}_{i}\left(\xi \right)\leq \frac{\left(\beta_{T}+\left(2\alpha_{D}+\alpha_{A}\right)kA{\bar{S}}_{o}\right)}{\gamma }.$$
  In addition,
  $$\frac{d(\bar{V}\left(\xi \right))}{d\xi }=\frac{N\lambda_{{\bar{T}}_{i}}}{c}{\bar{T}}_{i}\left(\xi \right)-\frac{c_{v}}{c}\bar{V}\left(\xi \right)+\frac{m^{2}}{c}\frac{d^{2}\left(d_{4}\bar{V}\right)}{d\xi^{2}}$$
  by the second derivative test theory
  $$\frac{d^{2}\left(d_{4}\bar{V}\left(\xi \right)\right)}{d\xi^{2}}<0,$$
  $$\frac{d(\bar{V}\left(\xi \right))}{d\xi }\leq \frac{N\lambda_{{\bar{T}}_{i}}}{c}\frac{\left(\beta_{T}+\left(2\alpha_{D}+\alpha_{A}\right)kA{\bar{S}}_{o}\right)}{\gamma }-\frac{c_{v}}{c}\bar{V}\left(\xi \right)$$
  $$\underset{\xi \to \infty }{\text{lim}}\;\text{sup}\frac{d(\bar{V}\left(\xi \right))}{d\xi }\leq \frac{N\lambda_{{\bar{T}}_{i}}}{c_{v}}\frac{\left(\beta_{T}+\left(2\alpha_{D}+\alpha_{A}\right)kA{\bar{S}}_{o}\right)}{\gamma }.$$
  $$\bar{V}\left(\xi \right)\leq \frac{N\lambda_{{\bar{T}}_{i}}}{c_{v}}\frac{\left(\beta_{T}+\left(2\alpha_{D}+\alpha_{A}\right)kA{\bar{S}}_{o}\right)}{\gamma }.$$
2. Basic reproduction number.
  In the case of infectious disease models the basic reproduction number *R*_*o*_, is a crucial factor that determines the likelihood of outbreaks in a population. *R*_*o*_ is a mathematical threshold for the stability of a disease-free equilibrium and is connected to the epidemic peak and final magnitude [27, 28].
  Let *Q* = (*T*_*i*_, *S*, *T*, *V*). The model (1) can be rewritten as $Q^{\prime }=X\left(Q\right)-Y\left(Q\right)$, where
  $$Y\left(Q\right)=(\begin{array}{c} k_{T}TV\\ 0\\ 0\\ 0 \end{array}),$$
  and
  $$X\left(Q\right)=(\begin{array}{c} \lambda_{T_{i}}T_{i}\\ (\delta_{S}-k\left(\alpha_{S}-\alpha_{D}\right))S\\ -\beta_{T}+d_{T}T-\left(2\alpha_{D}+\alpha_{A}\right)kAS-r_{T}T(1-\frac{T}{T_{max}})+k_{T}TV\\ -N\lambda_{T_{i}}T_{i}+c_{v}V \end{array}).$$
  Now, evaluating the Jacobean of *Q* at *P*_0_, we have
  $$J\left(Y\left(P_{o}\right)\right)=(\begin{array}{cccc} 0 & 0 & 0 & k_{T}T_{o}\\ 0 & 0 & 0 & 0\\ 0 & 0 & 0 & 0\\ 0 & 0 & 0 & 0 \end{array}).$$
  Also,
  $$J\left(X\left(P_{o}\right)\right)=(\begin{array}{cccc} \lambda_{T_{i}} & 0 & 0 & 0\\ 0 & \delta_{s}-k\left(\alpha_{s}-\alpha_{D}\right) & 0 & 0\\ 0 & -Ak(\alpha_{A}+2\alpha_{D}) & d_{T}-r_{T}(1-\frac{T_{o}}{T_{\text{max}}})+\frac{r_{T}T_{o}}{T_{\text{max}}} & k_{T}T_{o}\\ -N\lambda_{T_{i}} & 0 & 0 & c_{v} \end{array}).$$
  $$=(\begin{array}{cccc} \frac{1}{\lambda_{T_{i}}} & 0 & 0 & 0\\ 0 & \frac{1}{\delta_{S}+k\left(\alpha_{D}-\alpha_{S}\right)} & 0 & 0\\ NB & \frac{AkT_{\text{max}}\left(\alpha_{A}+2\alpha_{D}\right)}{\left(d_{T}T_{\text{max}}-T_{\text{max}}r_{T}+2r_{T}T_{0}\right)\left(\delta_{S}+k\left(\alpha_{D}-\alpha_{S}\right)\right)} & \frac{T_{\text{max}}}{d_{T}T_{\text{max}}-T_{\text{max}}r_{T}+2r_{T}T_{0}} & B\\ \frac{N}{c_{v}} & 0 & 0 & \frac{1}{c_{v}} \end{array}).$$
  where $B=-\frac{k_{T}T_{\text{max}}T_{0}}{c_{v}d_{T}T_{\text{max}}-c_{v}T_{\text{max}}r_{T}+2c_{v}r_{T}T_{0}}$
  Calculating the matrix for the next generation [29–32], we obtain
  $$\left(Y\left(P_{o}\right)\right)\left(X^{-1}\left(P_{o}\right)\right)=(\begin{array}{cccc} \frac{k_{T}NT_{o}}{c_{v}} & 0 & 0 & \frac{k_{T}T_{o}}{c_{v}}\\ 0 & 0 & 0 & 0\\ 0 & 0 & 0 & 0\\ 0 & 0 & 0 & 0 \end{array}).$$
  Then, the basic reproduction number *R*_*o*_ is given by the spectrum radius of the matrix $Y\left(P_{o}\right))\left(X^{-1}\left(P_{o}\right)\right)$,
  $$R_{o}=\rho \left(\left(Y\left(P_{o}\right)\right)\left(X^{-1}\left(P_{o}\right)\right)\right)=\frac{k_{T}NT_{o}}{c_{v}}.$$
  Because equilibrium points have biological significance, all of its constituents must be nonnegative and exist. *P*_0_ is always present, whereas P_*_ is present only if $d_{T}<\frac{\beta_{T}R_{o}}{T_{o}}$.
3. Equilibrium points
  The model (1) has two equilibrium points: *p*_0_(*S*, *T*, *T*_*i*_, *V*) = (0, *T*_*o*_, 0, 0) which is stable for all values of *R*_*o*_ and $p^{*}\left(S,T,T_{i},V\right)=\left(0,T^{*},\frac{c_{v}}{\lambda_{T_{i}}\text{N}}V^{*},V^{*}\right)$ which is stable only when *R*_*o*_ > 1 and $d_{T}<\frac{\beta_{T}R_{o}}{T_{o}}$.
  where
  $$T_{o}=\frac{T_{max}}{2r_{T}}(\left(r_{T}-d_{T}\right)+\sqrt{(r_{T}-{d_{T})}^{2}+\frac{4\beta_{T}r_{T}}{T_{max}}})$$
  $$T^{*}=\frac{c_{v}}{k_{T}\text{N}}$$
  $$V^{*}=\frac{-d_{T}-\frac{r_{T}T_{o}}{R_{o}T_{max}}+r_{T}+\frac{\beta_{T}R_{o}}{T_{o}}}{k_{T}}.$$
  If *R*_*o*_ > 1, then $\frac{T_{o}}{R_{o}T_{max}}<1$ since *T*_*max*_ > *T*_*o*_. Thus, P* > 0 if $d_{T}<\frac{\beta_{T}R_{o}}{T_{o}}$. It is clear that the equilibrium point *P*_*o*_ is positive for all values.
4. Local Stability
  **Theorem 2** *If R*_*o*_ < 1, *then the free infection equilibrium point P*_*o*_ *is locally asymptotically stable, if R*_*o*_ ≥ 0, *then P*_*o*_ *is unstable*
**Proof**
  We find the Jacobean matrix of the system (1) as follows
  $$J=(\begin{array}{cccc} k\left(\alpha_{s}-\alpha_{D}\right)-\delta_{s} & 0 & 0 & 0\\ (2\alpha_{D}+\alpha_{A})kA & -d_{T}-k_{T}V+r_{T}(1-\frac{T}{T_{\text{max}}})-\frac{r_{T}T}{T_{\text{max}}} & 0 & -k_{T}T\\ 0 & k_{T}V & -\lambda_{T_{i}} & k_{T}T\\ 0 & 0 & \lambda_{T_{i}}N & -c_{v}. \end{array})$$
  Then, we evaluate the free infection equilibrium point *P*_0_ which is *J*(*P*_0_)
  $$J\left(P_{o}\right)=(\begin{array}{cccc} k\left(\alpha_{s}-\alpha_{D}\right)-\delta_{s} & 0 & 0 & 0\\ (2\alpha_{D}+\alpha_{A})kA & -d_{T}+r_{T}(1-\frac{T_{o}}{T_{\text{max}}})-\frac{r_{T}T_{o}}{T_{\text{max}}} & 0 & -k_{T}T_{o}\\ 0 & 0 & -\lambda_{T_{i}} & k_{T}T_{o}\\ 0 & 0 & \lambda_{T_{i}}N & -c_{v}. \end{array})$$
  The matrix *J*(*P*_*o*_) has negative real part of eigenvalues if det(*J*(*P*_*o*_)) < 0, thus we have
  $$\begin{array}{cc} \text{det}(J\left(P_{o}\right)-\lambda I) & =\left(\left(k\left(\alpha_{s}-\alpha_{D}\right)-\delta_{s}\right)-\lambda \right)\left(-E_{o}-\lambda \right)(\lambda^{2}+\left(c_{v}+\lambda_{T_{i}}\right)\lambda +(\lambda_{T_{i}}c_{v}\\ & -N\lambda_{T_{i}}kT_{o})) \end{array}$$
  where
  $$E_{o}=d_{T}-r_{T}(1-\frac{T_{o}}{T_{\text{max}}})+\frac{r_{T}T_{o}}{T_{\text{max}}}=\frac{\beta_{T}}{T_{o}}+\frac{rT_{o}}{T_{max}}>0,$$
  from the first equation in the model. The λ_1_ = (*k*(*α*_*s*_ − *α*_*D*_) − *δ*_*s*_) < 0 from the condition for the solutions of *S* and λ_2_ = −*E*_*o*_ < 0. The two eigenvalues λ_3,4_ have negative real portions if and only if $\lambda_{T_{i}}c_{v}-N\lambda_{T_{i}}kT_{o}>0$ i.e., $R_{0}=\frac{Nk_{T}T_{o}}{c_{v}}<1$. If *R*_0_ = 1, *J*(*P*_0_) has one positive eigenvalue which is 0 and it is simple. If *R*_0_ > 1, the eigenvalues of *J*(*P*_0_) are positive.
  **Theorem 3** *The chronic-infection equilibrium* P* *is locally asymptotically stable if* *R*_0_ > 1 *and* $d_{T}<\frac{\beta_{T}R_{o}}{T_{o}}$.
**proof**
  The Jacobean matrix of the system (1) at P* is
  $$J\left(P^{*}\right)=(\begin{array}{cccc} k\left(\alpha_{s}-\alpha_{D}\right)-\delta_{s} & 0 & 0 & 0\\ (2\alpha_{D}+\alpha_{A})kA & -d_{T}-k_{T}V^{*}+r_{T}(1-\frac{T^{*}}{T_{\text{max}}})-\frac{r_{T}T^{*}}{T_{\text{max}}} & 0 & -k_{T}T^{*}\\ 0 & k_{T}V^{*} & -\lambda_{T_{i}} & k_{T}T^{*}\\ 0 & 0 & \lambda_{T_{i}}N & -c_{v} \end{array})$$
  where
  $$E_{*}=d_{T}+k_{T}V^{*}+r_{T}(1-\frac{T^{*}}{T\text{max}})-\frac{r_{T}T^{*}}{T\text{max}}=\frac{\beta_{T}}{T^{*}}+\frac{rT^{*}}{T_{max}}>0.$$
  The characteristic polynomial of *J*(P*) is
  $$P\left(\lambda \right)=\left(k\left(\alpha_{s}-\alpha_{D}\right)-\delta_{s}-\lambda \right)(\lambda^{3}+\left(E_{*}+\lambda_{T_{i}}+c_{v}\right)\lambda^{2}+E_{*}\left(\lambda_{T_{i}}+c_{v}\right)\lambda +k_{T}\lambda_{T_{i}}c_{v}V^{*})$$
  Note that λ_1_ = (*k*(*α*_*s*_ − *α*_*D*_) − *δ*_*s*_) < 0 from the condition for the solutions of *S*. The eigenvalues λ_2,3,4_ have negative real parts if and only if, according to the Routha Hurwitz criteria.
  $$△=\left(E_{*}+\lambda_{T_{i}}+c_{v}\right)E_{*}\left(\lambda_{T_{i}}+c_{v}\right)-k_{T}\lambda_{T_{i}}c_{v}V^{*}>0$$
  Note by substituting E_*_ into △, the negative term $-k_{T}\lambda_{T_{i}}c_{v}V^{*}$ is canceled and △ > 0. Therefore the point P* is locally asymptotically stable if $d_{T}<\frac{\beta_{T}R_{o}}{T_{o}}$.
5. Global Stability
  **Theorem 4** *The equilibrium point P*_*o*_ *is globally asymptotically stable if and only if* *R*_*o*_ < 1, $\beta_{T}<d_{T}T_{o}+r_{T}T_{o}(\frac{T_{o}}{T_{max}}-1)$.
**Proof**
  We use the comparison theorem to prove the global stability of the disease free equilibrium. The rate of change of the variables (*T*_*i*_, *T*, *S*, *V*) of the system.
  Let
  $$Z=(\begin{array}{cccc} \lambda_{T_{i}} & 0 & 0 & 0\\ 0 & d_{T} & -Ak(\alpha_{a}+2\alpha_{d}) & 0\\ 0 & 0 & \delta_{s}-k\left(\alpha_{s}-\alpha_{d}\right) & 0\\ -N\lambda_{T_{i}} & 0 & 0 & c_{v} \end{array}),$$
  $$L=(\begin{array}{cccc} 0 & 0 & 0 & k_{T}T_{o}\\ 0 & -\frac{r_{T}T_{o}}{T_{\text{max}}}+\frac{r_{T}T_{o}}{T}+\frac{\beta_{T}}{T^{2}} & 0 & -k_{T}T_{o}\\ 0 & 0 & 0 & 0\\ 0 & 0 & 0 & 0 \end{array}).$$
  Thus, we have
  $$\begin{array}{cc} c\left({T_{i}}^{\prime },T^{\prime },S^{\prime },V^{\prime }\right)-m^{2}\left({d_{3}T_{i}}^{\prime \prime },d_{1}T^{\prime \prime },d_{1}S^{\prime \prime },d_{4}V^{\prime \prime }\right)= & \left(L-Z\right)\left(T_{i},T,S,V\right)-\left(1-\frac{T}{T_{o}}\right)\\ & L(T_{i},T,S,V) \end{array}$$
  If *T* → *T*_*o*_, then we have
  $$c\left({T_{i}}^{\prime },T^{\prime },S^{\prime },V^{\prime }\right)-m^{2}\left({d_{3}T_{i}}^{\prime \prime },d_{1}T^{\prime \prime },d_{1}S^{\prime \prime },d_{4}V^{\prime \prime }\right)\leq \left(L-Z\right)\left(T_{i},T,S,V\right)$$
  $$L-Z=(\begin{array}{cccc} -\lambda_{T_{i}} & 0 & 0 & k_{T}T_{o}\\ 0 & -d_{T}-\frac{r_{T}T_{o}}{T_{\text{max}}}+r_{T}+\frac{\beta_{T}}{T_{o}} & Ak(\alpha_{a}+2\alpha_{d}) & -k_{T}T_{o}\\ 0 & 0 & k\left(\alpha_{s}-\alpha_{d}\right)-\delta_{s} & 0\\ \lambda_{T_{i}}N & 0 & 0 & -c_{v} \end{array})$$
  We have the eigenvalues
  $$\begin{array}{cc} \lambda_{1} & =\frac{-d_{T}T_{max}T_{o}+\beta_{T}T_{max}+T_{max}r_{T}T_{o}-r_{T}T_{o}^{2}}{T_{max}T_{o}},\\ \lambda_{2} & =k\left(\alpha_{s}-\alpha_{d}\right)-\delta_{s},\\ \lambda_{3,4} & =\frac{-T_{max}T_{o}\left(c_{v}+\lambda_{T_{i}}\right)\pm T_{max}T_{o}\sqrt{c_{v}^{2}-2c_{v}\lambda_{T_{i}}+4k_{T}\lambda_{T_{i}}NT_{o}+\lambda_{T_{i}}^{2}}}{2T_{max}T_{o}}. \end{array}$$
  Since *R*_*o*_ < 1, which means *k*_*T*_*NT*_*o*_ < *c*_*v*_, then
  $$\lambda_{3}\leq \frac{-T_{max}T_{o}\left(c_{v}+\lambda_{T_{i}}\right)+T_{max}T_{o}\left(c_{v}+\lambda_{T_{i}}\right)}{2T_{max}T_{o}}=0,$$
  $$\lambda_{4}\leq \frac{-T_{max}T_{o}\left(c_{v}+\lambda_{T_{i}}\right)-T_{max}T_{o}\left(c_{v}+\lambda_{T_{i}}\right)}{2T_{max}T_{o}}=-\left(c_{v}+\lambda_{T_{i}}\right).$$
  Thus, the eigenvalues λ_3,4_ have a negative real part and λ_1_ has a negative real part if $\beta_{T}<d_{T}T_{o}+r_{T}T_{o}(\frac{T_{o}}{T_{max}}-1)$. From the condition of local stability of *P*_*o*_, *k*(*α*_*s*_ − *α*_*d*_) − *δ*_*s*_ < 0, thus λ_2_ has a negative real part. So, *P*_*o*_ is globally asymptotically stable.
  **Theorem 5** *The equilibrium point* P* *is globally asymptotically stable if* (2*α*_*D*_ + *α*_*A*_)*kA* < *k*(*α*_*D*_ − *α*_*s*_) + *δ*_*s*_), $d_{T}>r_{T}\left(\left(\frac{T^{*}}{T_{max}}\right)+1\right)$ *and* $r_{T}<\frac{2\beta_{T}c_{v}V^{*}T_{max}}{NT{}^{*}{}^{2}}$.
**Proof**
  Define the Lyapunov function as follows:
  $$L\left(S,T,T_{i},V\right)=S+\left(T-T^{*}\;\text{ln}\;T\right)+\left(T_{i}-T_{i}^{*}\;\text{ln}\;T_{i}\right)+\frac{1}{N}\left(V-V^{*}\text{ln}V\right)$$
  Clearly, *L* is a positive definite function. and *L*′ is
  $$\begin{array}{cc} L^{\prime } & =\left(k\left(\alpha_{s}-\alpha_{D}\right)-\delta_{s}\right)S+(1-\frac{T^{*}}{T})(\beta_{T}-d_{T}T+\left(2\alpha_{D}+\alpha_{A}\right)kAS+\\ & r_{T}T(1-\frac{T}{T_{max}})-k_{T}TV)+(1-\frac{T_{i}^{*}}{T_{i}})(k_{T}TV-\lambda_{T_{i}}T_{i})+\\ & \frac{1}{N}(1-\frac{V^{*}}{V})(N\lambda_{T_{i}}T_{i}-c_{v}V). \end{array}$$
  Using the equilibrium equations at P* gives
  $$\beta_{T}=d_{T}T^{*}-r_{T}T^{*}(1-\frac{T^{*}}{T_{max}})+k_{T}T^{*}V^{*}$$
  $$k_{T}T^{*}V^{*}-\lambda_{T_{i}}T_{i}^{*}=0$$
  $$N\lambda_{T_{i}}T_{i}^{*}-c_{v}V^{*}=0$$
  Thus, we have the follows
  $$L^{\prime }=C_{S}S+C_{T}T+C_{V}V+C_{T^{2}}T^{2}+C_{TV}TV+C_{\frac{S}{T}}\frac{S}{T}+C_{\frac{1}{T}}\frac{1}{T}+C_{\frac{TV}{T_{i}}}\frac{TV}{T_{i}}+C_{\frac{T_{i}}{V}}\frac{T_{i}}{V}+C_{o}$$
  where
  $$\begin{array}{cc} C_{S} & =\left(k\left(\alpha_{s}-\alpha_{D}\right)-\delta_{s}\right)+\left(2\alpha_{D}+\alpha_{A}\right)kA,\\ C_{T} & =-d_{T}+r_{T}\frac{T^{*}}{T_{max}}+r_{T},\\ C_{V} & =k_{T}T^{*}-\frac{1}{N}c_{v},\\ C_{T^{2}} & =-r_{T}\frac{1}{T_{max}},\\ C_{TV} & =-k_{T}+k_{T},\\ C_{\frac{S}{T}} & =-\left(2\alpha_{D}+\alpha_{A}\right)kAT^{*},\\ C_{\frac{1}{T}} & =-T^{*}\beta_{T},\\ C_{\frac{TV}{T_{i}}} & =-k_{T}T^{*},\\ C_{\frac{T_{i}}{V}} & =-\frac{1}{N}N\lambda_{T_{i}}V^{*},\\ C_{o} & =\beta_{T}+d_{T}T^{*}-r_{T}T^{*}+\lambda_{T_{i}}T_{i}^{*}+\frac{1}{N}c_{v}V^{*}. \end{array}$$
  It is obvious that $C_{T^{2}},C_{TV},C_{\frac{S}{T}},C_{\frac{1}{T}},C_{\frac{TV}{T_{i}}},C_{\frac{T_{i}}{V}}$ are of negative value. Using the equilibrium equations at P* gives
  $$C_{V}=k_{T}T^{*}-\frac{1}{N}c_{v}=(\frac{\lambda_{T_{i}}T^{*}}{V^{*}})-\frac{1}{N}(\frac{N\lambda_{T_{i}}T^{*}}{V^{*}})=0$$
  From the condition of the solution of *S*, we know (*k*(*α*_*s*_ − *α*_*D*_) − *δ*_*s*_) < 0, thus *C*_*S*_ < 0 if (2*α*_*D*_ + *α*_*A*_)*kA* < *k*(*α*_*D*_ − *α*_*s*_) + *δ*_*s*_) and *C*_*T*_ < 0 if $d_{T}>r_{T}\left(\left(\frac{T^{*}}{T_{max}}\right)+1\right)$
  By using the equilibrium equations at P*, *C*_*o*_ becomes
  $$\begin{array}{cc} C_{o} & =\beta_{T}+d_{T}T^{*}-r_{T}T^{*}+\lambda_{T_{i}}T_{i}^{*}+\frac{1}{N}c_{v}V^{*}\\ & =2\beta_{T}-r_{T}T^{*}(\frac{T^{*}}{T_{max}})+(\frac{c_{v}V^{*}}{N}) \end{array}$$
  Thus, *C*_0_ < 0 if $r_{T}<\frac{2\beta_{T}c_{v}V^{*}T_{max}}{NT{}^{*}{}^{2}}$. Hence, P* is globally asymptotically stable.

## 1 Analytical solution for system of PDEs

The tanh-expansion technique was introduced by Malfliet and is a powerful and dependable method for dealing with a variety of nonlinear dispersive and dissipative equations. This technique has been extended in several ways and is now widely utilized. Because all derivatives of a tanh function are represented by a tanh itself, Malfliet adapted the tanh approach by introducing tanh as a new variable to prevent complication. After that, a simple analysis may be performed to ensure that the method is applicable to a wide range of nonlinear equations. The tanh technique in its standard form, as presented by Malfliet, employed in this study. The tanh approach is based on the premise that traveling wave solutions can be described as a succession of tanh functions. The key steps of the standard tanh technique [28] are as follows:

1. Using traveling wave transformation *ξ* = *ct* + *mx*^2^, *m* is the frequency number, to transfer the system of PDEs to the system of ODEs and we obtain Eq (2).
2. Assume $\bar{S}\left(\xi \right)=\alpha_{1}U\left(\xi \right)$, $\bar{T}\left(\xi \right)=\alpha_{2}U\left(\xi \right)$, ${\bar{T}}_{i}\left(\xi \right)=\alpha_{3}U\left(\xi \right)$, $\bar{V}\left(\xi \right)=\alpha_{4}U\left(\xi \right)$, then, We get an equation in *U* and its derivative
  $$P=(U,U^{\prime },U^{\prime \prime },\cdots )=0.$$
3. Introduce of a new independent variable, *Y* = tanh(*Mξ*), and assume $U\left(\xi \right)=Z\left(Y\right)=\sum_{i=0}^{n}a_{i}Y^{i}$, where M is a positive integer and no boundary conditions are imposed. Then, the derivatives are changed as follows:
  $$\begin{array}{cc} \frac{dU(\xi )}{d_{\xi }} & =\mu \left(1-Y^{2}\right)\frac{dZ(Y)}{dY},\\ \frac{d^{2}U\left(\xi \right)}{d_{\xi }^{2}} & =\mu^{2}\left(1-Y^{2}\right)\left(\left(-2Y\frac{dZ(Y)}{dY}\right)+\left(1-Y^{2}\right)\left(\frac{d^{2}Z\left(Y\right)}{dY^{2}}\right)\right), \end{array}$$
  where other derivatives can be obtained in the same way. Thus, they are entirely dependent on Y [29, 30].
4. Using the balance between the highest order linear terms in the resulting equation and the highest order nonlinear terms, we determine the parameter *n*.
5. All coefficients *Y*^*i*^ are equated to zero to obtain *α*_*i*_’s and *a*_*i*_’s.

Following steps (1), (2), the system of model (1) is reduced to

$$\begin{array}{cc} {\bar{S}}^{\prime }\left(\xi \right) & =\frac{1}{c}(\left(k\left(\alpha_{s}-\alpha_{D}\right)-\delta_{s}\right)\left(\alpha_{1}U\left(\xi \right)\right)+m^{2}d_{1}(\alpha_{1}U^{\prime \prime }\left(\xi \right))\\ {\bar{T}}^{\prime }\left(\xi \right) & =\frac{1}{c}(\beta_{T}-d_{T})\left(\alpha_{2}U\left(\xi \right)\right)+\left(2\alpha_{D}+\alpha_{A}\right)kA\left(\alpha_{1}U\left(\xi \right)\right)+\\ & r_{T}\left(\alpha_{2}U\left(\xi \right)\right)(1-\frac{\alpha_{2}U\left(\xi \right)}{U_{max}})-k_{T}\left(\alpha_{3}U\left(\xi \right)\right)\left(\alpha_{4}U\left(\xi \right)\right)+m^{2}d_{2}\left(\alpha_{2}U^{\prime \prime }\left(\xi \right)\right)\\ {\bar{T}}_{i}^{\prime }\left(\xi \right) & =\frac{1}{c}(k_{T}\left(\alpha_{2}U\left(\xi \right)\right)\left(\alpha_{4}U\left(\xi \right)\right)-\lambda_{T_{i}}\left(\alpha_{3}U\left(\xi \right)\right)+m^{2}d_{3}\left(\alpha_{3}U^{\prime \prime }\left(\xi \right)\right))\\ {\bar{V}}^{\prime }\left(\xi \right) & =\frac{1}{c}(N\lambda_{T_{i}}\left(\alpha_{3}U\left(\xi \right)\right)-c_{v}\left(\alpha_{4}U\left(\xi \right)\right)+m^{2}d_{4}\left(\alpha_{4}U^{\prime \prime }\left(\xi \right)\right)) \end{array}$$

Then the sum of all the equations $\left({\bar{S}}^{\prime }\left(\xi \right)+{\bar{T}}^{\prime }\left(\xi \right)+{\bar{T}}_{i}^{\prime }\left(\xi \right)+{\bar{V}}^{\prime }\left(\xi \right)\right)$, yields to then, we can rewrite it as:

$$\begin{array}{c} \left(k\left(\alpha_{s}-\alpha_{D}\right)-\delta_{s}\right)\left(\alpha_{1}U\left(\xi \right)\right)+\beta_{T}-d_{T}\left(\alpha_{2}U\left(\xi \right)\right)+\left(2\alpha_{D}+\alpha_{A}\right)kA\left(\alpha_{1}U\left(\xi \right)\right)+\\ r_{T}\left(\alpha_{2}U\left(\xi \right)\right)\;(1-\frac{\alpha_{2}U\left(\xi \right)}{U_{max}})-\lambda_{T_{i}}\left(\alpha_{3}U\left(\xi \right)\right)+N\lambda_{T_{i}}\left(\alpha_{3}U\left(\xi \right)\right)-c_{v}\left(\alpha_{4}U\left(\xi \right)\right)\\ -c\left(\alpha_{1}+\alpha_{2}+\alpha_{3}+\alpha_{4}\right)U^{\prime }\left(\xi \right)+m^{2}\left(d_{1}\alpha_{1}+d_{2}\alpha_{2}+d_{3}\alpha_{3}+d_{4}\alpha_{4}\right)U^{\prime \prime }\left(\xi \right)=0. \end{array}$$ (3)

Balancing the highest derivative term with highest order of the nonlinear terms, we find *n* = 2 and

$$\begin{array}{c} Z=a_{0}+a_{1}Y+a_{2}Y^{2},\;\frac{dZ}{dY}=a_{1}+2a_{2}Y,\;\frac{d^{2}Z}{dY^{2}}=2a_{2}. \end{array}$$

Substituting that into the ODE (4) gives

$$\begin{array}{c} b_{0}+b_{1}Y+b_{2}Y^{2}+b_{3}Y^{3}+b_{4}Y^{4}=0. \end{array}$$ (4)

where

$$\begin{array}{cc} b_{0} & =-a_{1}\alpha_{1}c\mu -a_{1}\alpha_{2}c\mu -a_{1}\alpha_{3}c\mu -a_{1}\alpha_{4}c\mu +2a_{2}\alpha_{1}c\mu Y^{3}+2a_{2}\alpha_{2}c\mu Y^{3}+2a_{2}\alpha_{3}c\mu Y^{3}+\\ & 2a_{2}\alpha_{4}c\mu Y^{3}+a_{1}\alpha_{1}c\mu Y^{2}+a_{1}\alpha_{2}c\mu Y^{2}+a_{1}\alpha_{3}c\mu Y^{2}+a_{1}\alpha_{4}c\mu Y^{2}-2a_{2}\alpha_{1}c\mu Y-2a_{2}\alpha_{2}c\mu Y-\\ & 2a_{2}\alpha_{3}c\mu Y-2a_{2}\alpha_{4}c\mu Y+2a_{2}\alpha_{2}d_{2}m^{2}\mu^{2}+6a_{2}\alpha_{2}d_{2}m^{2}\mu^{2}Y^{4}+2a_{1}\alpha_{2}d_{2}m^{2}\mu^{2}Y^{3}-\\ & 8a_{2}\alpha_{2}d_{2}m^{2}\mu^{2}Y^{2}-2a_{1}\alpha_{2}d_{2}m^{2}\mu^{2}Y+2a_{2}\alpha_{3}d_{3}m^{2}\mu^{2}+6a_{2}\alpha_{3}d_{3}m^{2}\mu^{2}Y^{4}+2a_{1}\alpha_{3}d_{3}m^{2}\mu^{2}Y^{3}-\\ & 8a_{2}\alpha_{3}d_{3}m^{2}\mu^{2}Y^{2}-2a_{1}\alpha_{3}d_{3}m^{2}\mu^{2}Y+2a_{2}\alpha_{4}d_{4}m^{2}\mu^{2}+6a_{2}\alpha_{4}d_{4}m^{2}\mu^{2}Y^{4}+2a_{1}\alpha_{4}d_{4}m^{2}\mu^{2}Y^{3}\\ & -8a_{2}\alpha_{4}d_{4}m^{2}\mu^{2}Y^{2}-2a_{1}\alpha_{4}d_{4}m^{2}\mu^{2}Y-\frac{1}{500}\alpha_{2}^{2}Y^{2}-0.01215\alpha_{1}Y+2.99\alpha_{2}Y+1.53\alpha_{3}Y-\\ & 3\alpha_{4}Y+0.39, \end{array}$$

$$\begin{array}{cc} b_{1} & =-2.\alpha_{2}(a_{2}c\mu +a_{1}d_{2}m^{2}\mu^{2}-1.495)-2a_{2}\alpha_{3}c\mu -2a_{2}\alpha_{4}c\mu -2.\alpha_{1}(a_{2}c\mu +0.006075)-\\ & 2a_{1}\alpha_{3}d_{3}m^{2}\mu^{2}-2a_{1}\alpha_{4}d_{4}m^{2}\mu^{2}+1.53\alpha_{3}-3\alpha_{4}, \end{array}$$

$$\begin{array}{cc} b_{2} & =a_{1}\alpha_{1}c\mu +a_{1}\alpha_{2}c\mu +a_{1}\alpha_{3}c\mu +a_{1}\alpha_{4}c\mu -8a_{2}\alpha_{2}d_{2}m^{2}\mu^{2}-8a_{2}\alpha_{3}d_{3}m^{2}\mu^{2}-8a_{2}\alpha_{4}d_{4}m^{2}\mu^{2}\\ & -\frac{\alpha_{2}^{2}}{500}, \end{array}$$

$$\begin{array}{cc} b_{3} & =2a_{2}\alpha_{1}c\mu +2a_{2}\alpha_{2}c\mu +2a_{2}\alpha_{3}c\mu +2a_{2}\alpha_{4}c\mu +2a_{1}\alpha_{2}d_{2}m^{2}\mu^{2}+2a_{1}\alpha_{3}d_{3}m^{2}\mu^{2}+\\ & 2a_{1}\alpha_{4}d_{4}m^{2}\mu^{2}, \end{array}$$

$$b_{4}=6a_{2}\alpha_{2}d_{2}m^{2}\mu^{2}+6a_{2}\alpha_{3}d_{3}m^{2}\mu^{2}+6a_{2}\alpha_{4}d_{4}m^{2}\mu^{2}.$$

Eq (4) is the fourth order in *Y* and by equating the constant *b*_*i*_ to zero, we obtain algebraic system. Solving this system by a Mathematica program gives the value of *α*_*i*_ and *a*_*i*_ and then we can obtain the solutions in terms of tanh-expansion.

## Discussion

**Fig 2:**
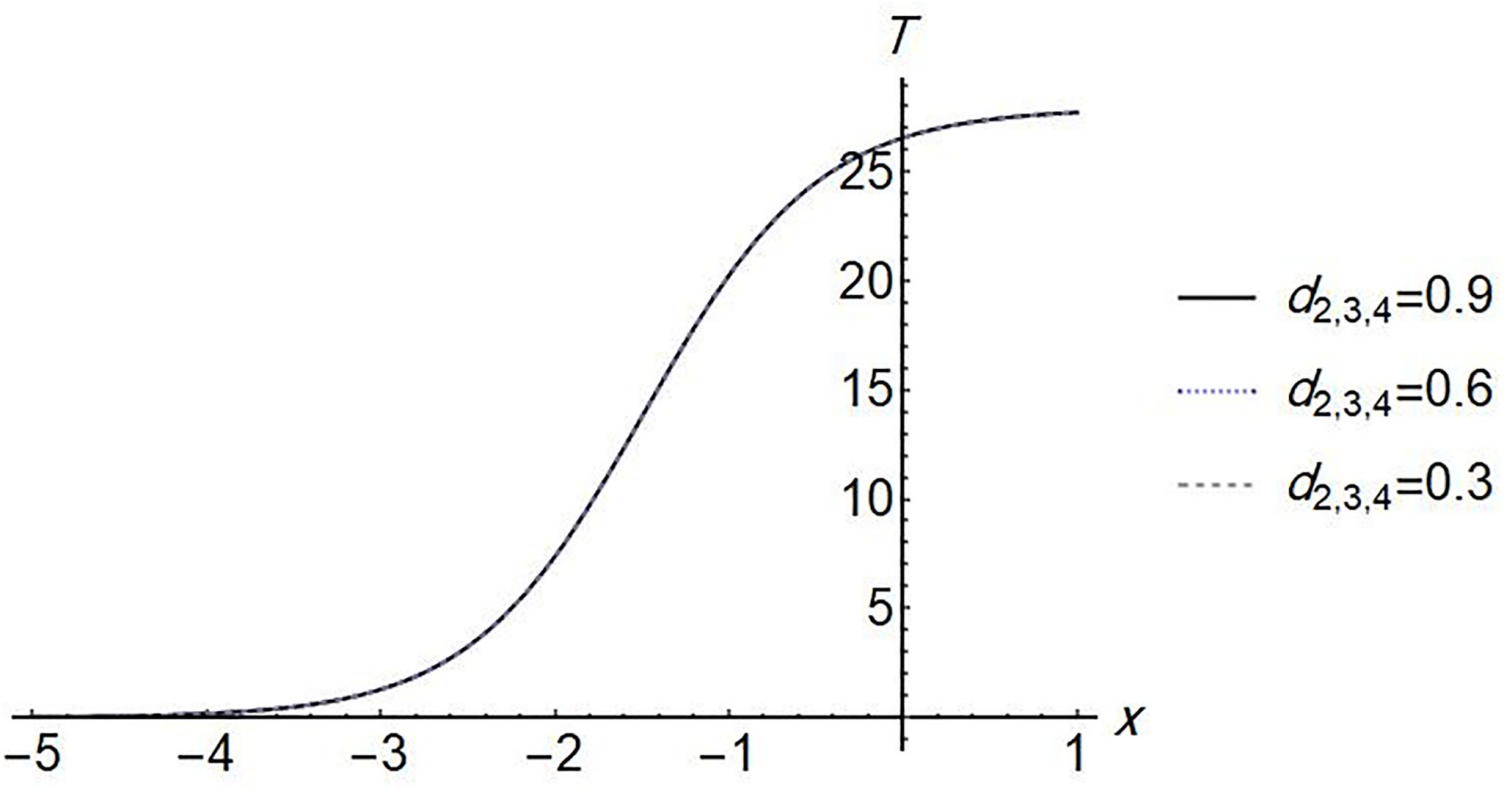
Plot of *T*(*x*, 2) for different values of all *d*_2_, *d*_3_, *d*_4_.

**Fig 3:**
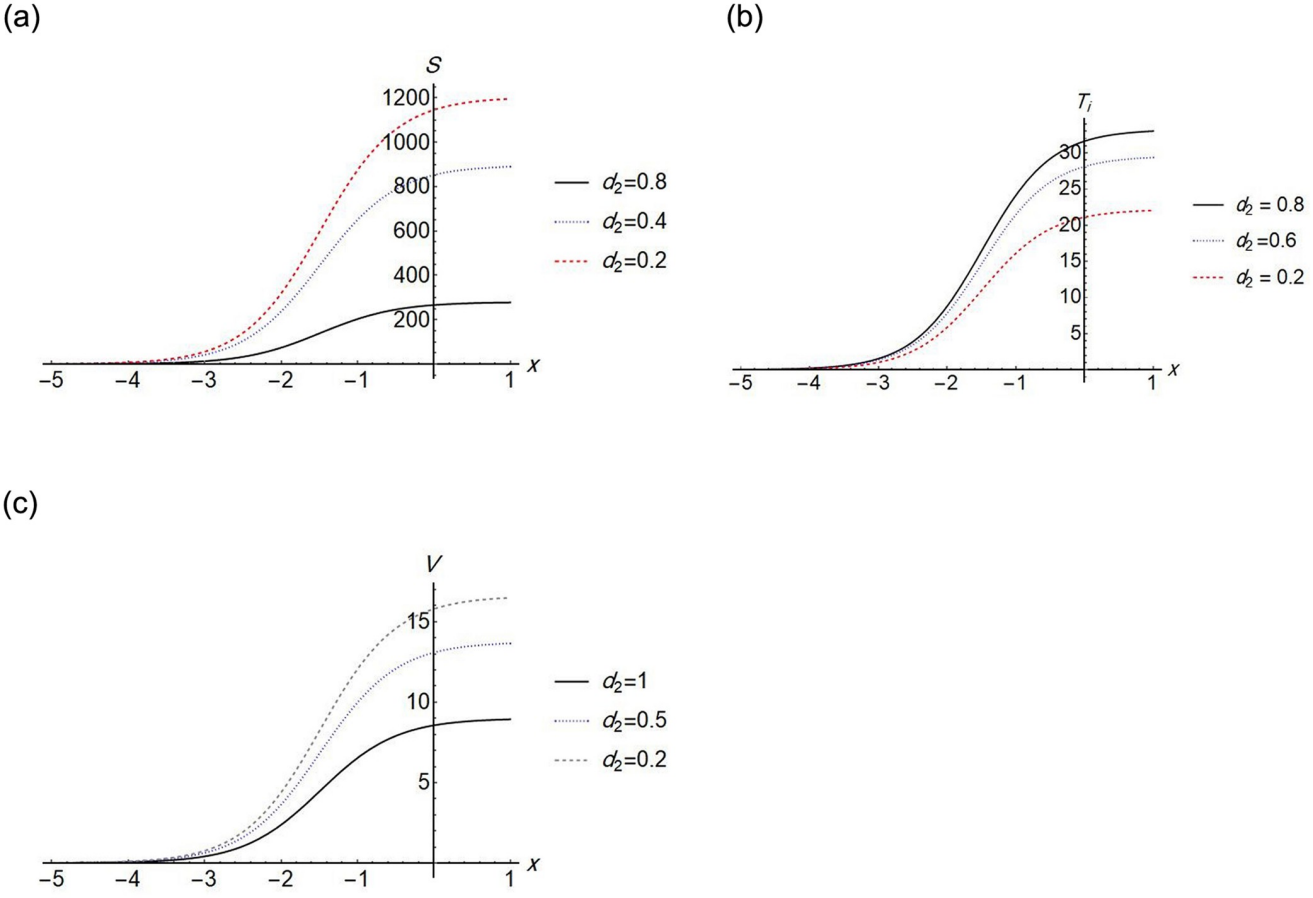
Plot of *S*(*x*, 2), *T*_*i*_(*x*, 2) and *V*(*x*, 2) for different values of *d*_2_.

**Fig 4:**
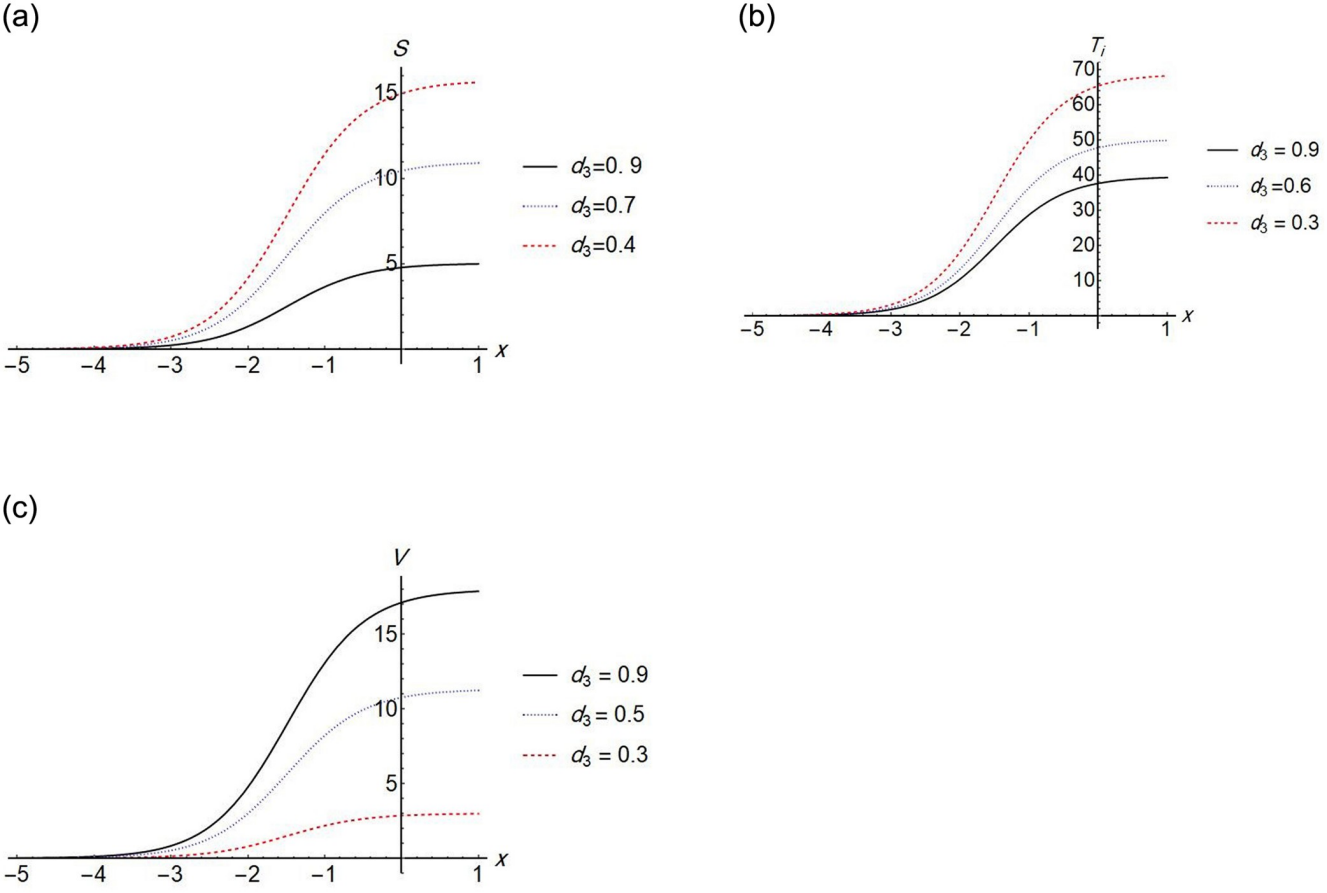
Plot of *S*(*x*, 2), *T*_*i*_(*x*, 2) and *V*(*x*, 2) for different values of *d*_3_.

**Fig 5:**
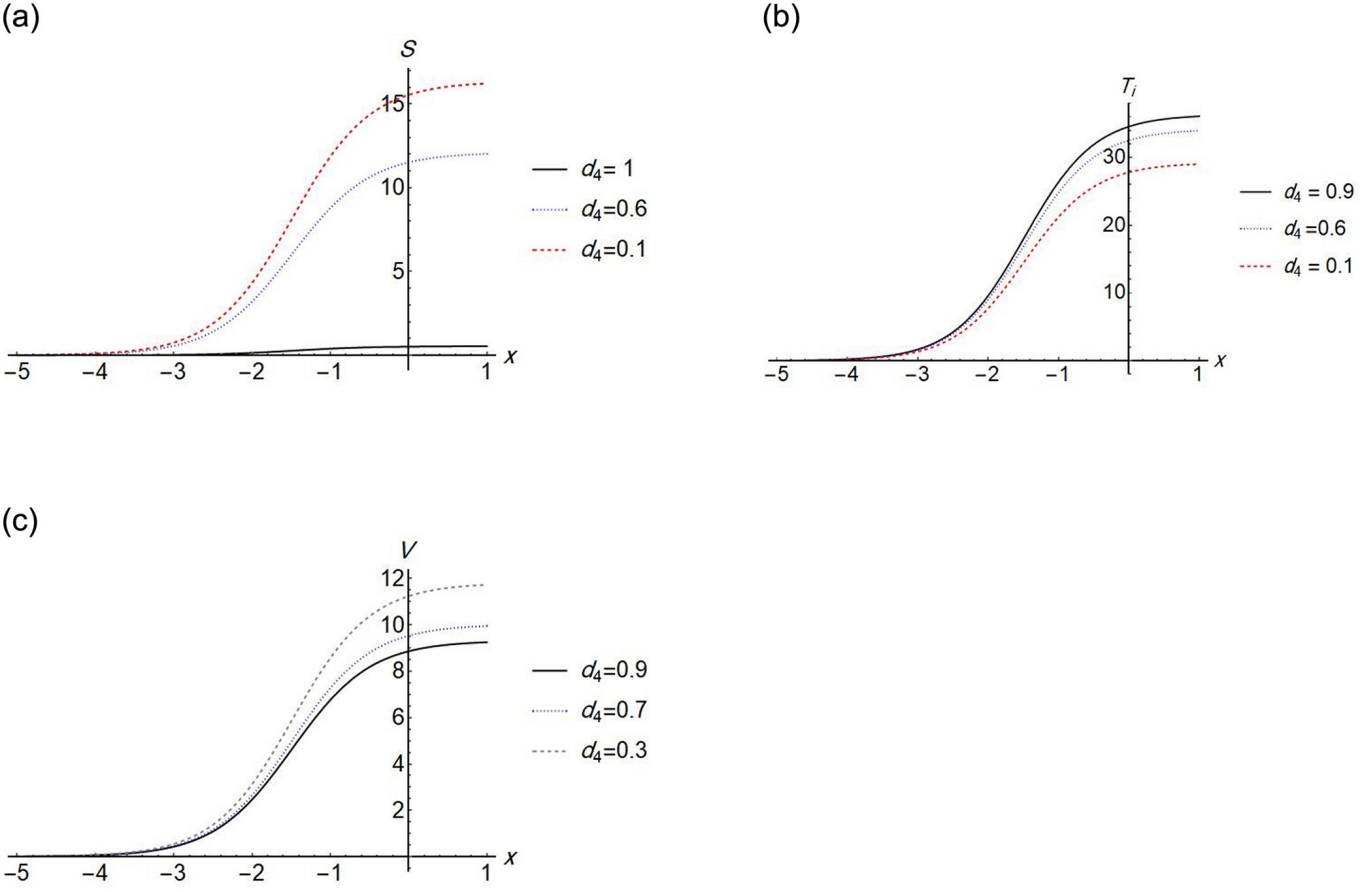
Plot of *S*(*x*, 2), *T*_*i*_(*x*, 2) and *V*(*x*, 2) for different values of *d*_4_.

**Fig 6:**
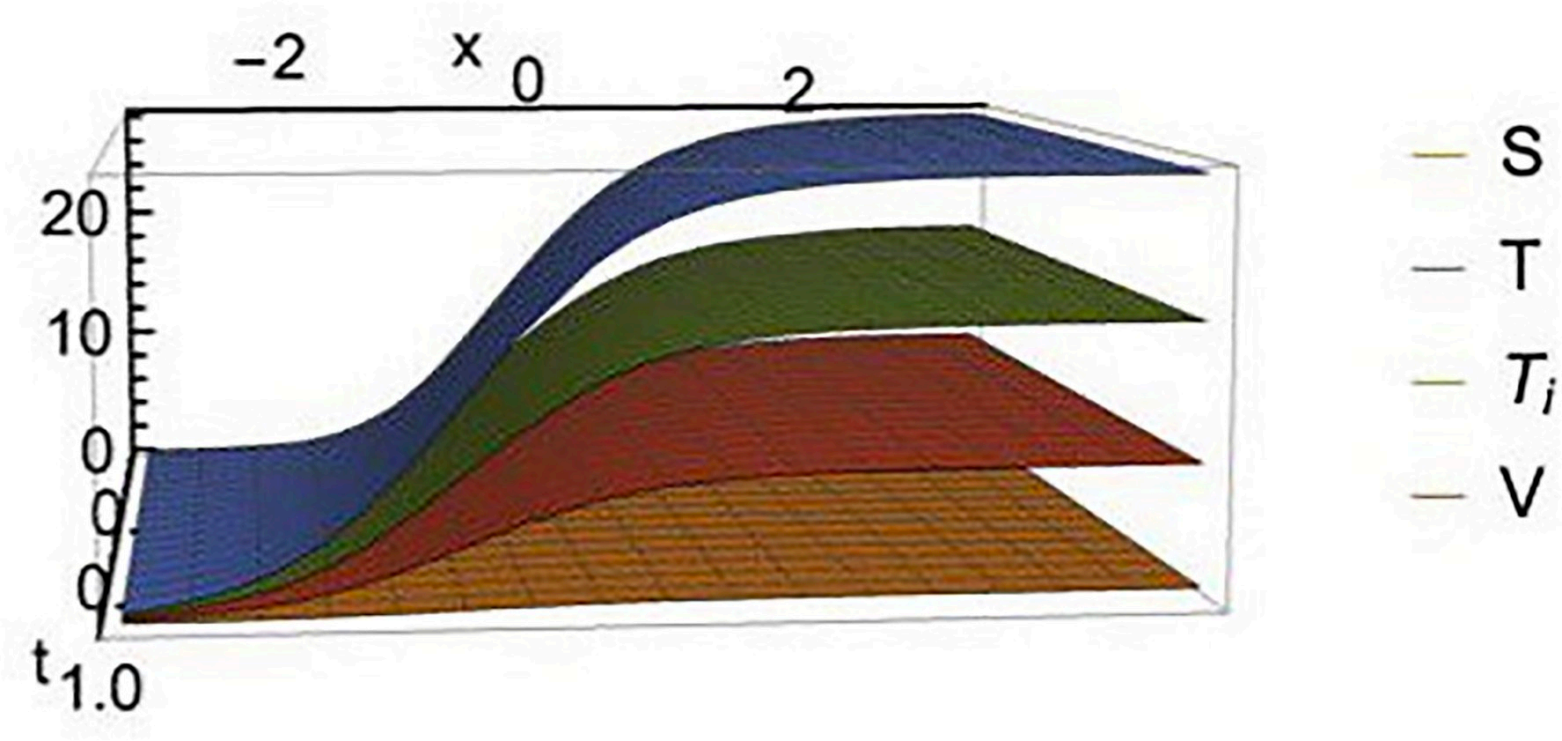
Plot of *S*(*x*, *t*), *T*(*x*, *t*), *T*_*i*_(*x*, *t*) and *V*(*x*, *t*).

We find that the solution exists when *d*_1_ = 0, which means that, the effect of the diffusion term of *S* disappears. The Figs 2–5 show the change in stem cell count, uninfected CD4^+^ T- cell, infected CD4^+^ T- cell, and virus density in the blood with respect of space (*x*). The Fig 2 shows that the uninfected *T*-cell is not affected by any change in any diffusion term. Fig 3 shows that as the *T*-cell spread in the space, the stem cell concentration and *T*_*i*_, increase while the virus decreases in the space. Fig 4 shows the change in *d*_3_ which is the diffusion term for infected cells, if *d*_3_ increases, *S* and *T*_*i*_ decrease, and *V* increases. Fig 5, represent the change in virus diffusion over the space, we see that the *S*, *T*_*i*_ and *V* increase when *d*_4_ increases. Fig 6 presents the solutions versus *x* and *t*.

## Conclusion

In this paper, we investigated a biological model describing the interaction of HIV-1 infected CD4^+^ T- cells with stem cell therapy. The equation of the model describes the rate of change of stem cell density, the second describes the rate of change of uninfected CD4^+^ T- cells, the third describes the rate of change of infected CD4^+^ T- cells, and the fourth, the rate of change of infected CD4^+^T- cells altered by the HIV-1 infection. The most important structural feature highlighted in this work is that we demonstrated that the solutions have bounded and positive. The model was found to exhibit stability. The results were obtained using the tanh-expansion method of HIV-1 infection with a mathematical model of CD4^+^ T- cells. It can be concluded that this method is very effective in obtaining analytical solutions within the sphere of modern epidemiology. For future work, the *diffusion effect* used to bolster a variety of mathematical models in the field of medicine. The intention is to incorporate the diffusion effect into PDEs mathematical model in future studies by the authors of this work under the optimal initial conditions, based on experimental study; in order to better understand the behaviour of the solutions and thus be in a position to pass on invaluable information to the doctors providing treatment to individuals.

## Supporting information

S1 Fig (JPG)

S2 Fig (JPG)

S3 Fig (JPG)

S4 Fig (JPG)
