# Supplementary figures and images for: Theoretical study of diffusive model of HIV-1 infection and its analytical solution
Source: PLoS One. 2023 Nov 10;18(11):e0283659. doi: 10.1371/journal.pone.0283659 (PMC10637654; doi:10.1371/journal.pone.0283659)

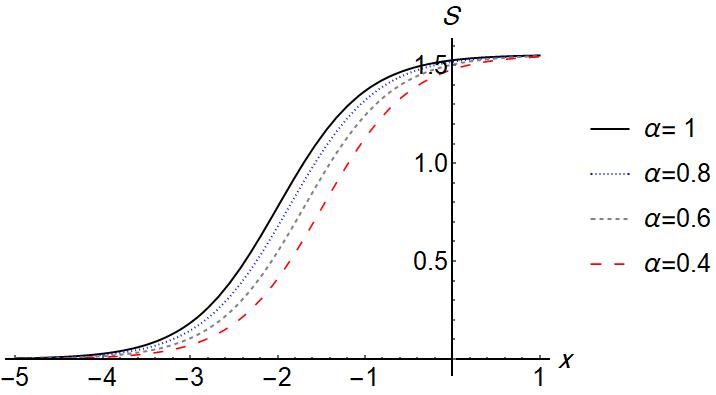

Supplement: S1 Fig — (JPG) [file pone.0283659.s001.jpg]

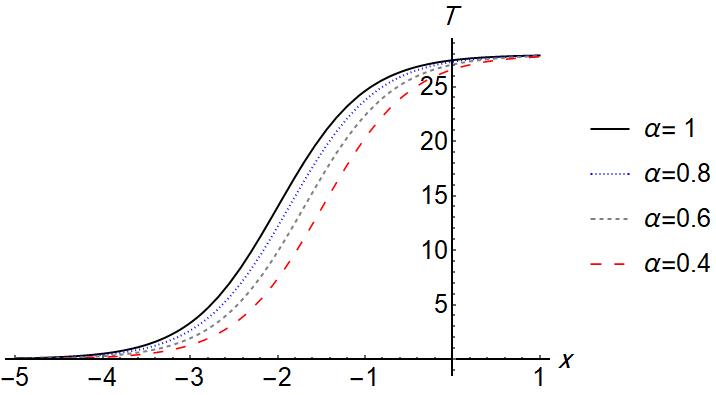

Supplement: S2 Fig — (JPG) [file pone.0283659.s002.jpg]

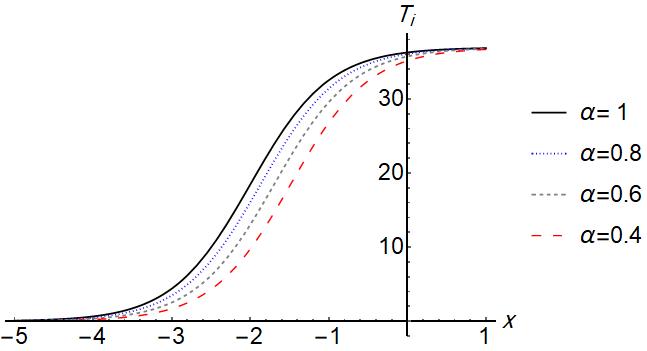

Supplement: S3 Fig — (JPG) [file pone.0283659.s003.jpg]

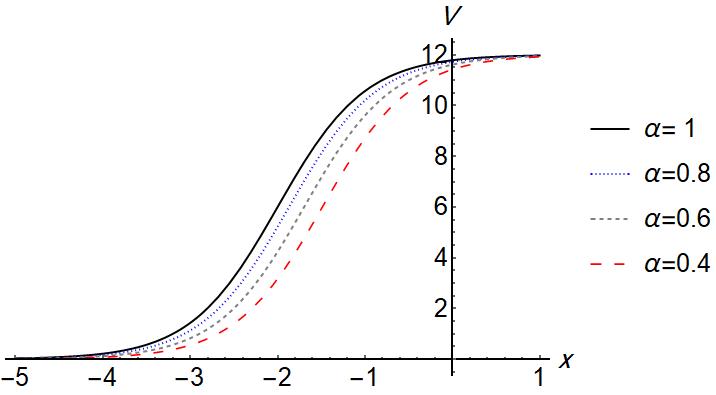

Supplement: S4 Fig — (JPG) [file pone.0283659.s004.jpg]
